# Electrode deactivation in cochlear implant users: impact on speech perception and objective measures

**DOI:** 10.1590/2317-1782/e20250290en

**Published:** 2026-07-27

**Authors:** Carlos Alberto Conceição Santana, Júlia Speranza Zabeu Fernandes, Luiz Fernando Manzoni Lourençone

**Affiliations:** 1 Hospital de Reabilitação de Anomalias Craniofaciais – HRAC, Universidade de São Paulo – USP - Bauru (SP), Brasil.; 2 Seção de Implante Coclear, Hospital de Reabilitação de Anomalias Craniofaciais – HRAC, Universidade de São Paulo – USP - Bauru (SP), Brasil.; 3 Departamento de Otorrinolaringologia, Faculdade de Medicina de Bauru, Universidade de São Paulo – USP - Bauru (SP), Brasil.

**Keywords:** Audiology, Hearing Loss, Cochlear Implant, Speech Perception, Evoked Potentials, Auditory Rehabilitation, Electrodes, Electrical Impedance

## Abstract

**Purpose:**

To verify whether electrode deactivation in cochlear implant users results in modifications in silent and noisy speech recognition, as well as to identify whether changes occur in electrical and physiological measurements.

**Methods:**

Retrospective study of medical records of 46 cochlear implant users, from three manufacturers, who underwent deactivation of at least one electrode. Pre- and post-deactivation data relating to programming records, free-field audiometry (500, 1,000, 2,000 and 4,000 Hz) and sentence recognition tests recorded in silence at 60 dB and with competing noise at +10 dB signal-to-noise ratio. Objective measurements included impedance telemetry, electrically elicited stapedial reflex (eSRT) and electrically evoked compound action potentials (ECAP). Statistical analyses used the Wilcoxon test, Spearman correlation, and chi-square test, with a significance level of 5%.

**Results:**

Statistically significant correlations were observed in the thresholds at 1,000 and 4,000 Hz in the 46 users studied, but without functional impact on overall averages or speech perception between pre- and post-deactivation assessments. Objective measures showed significant changes, including an increase in electrodes with altered impedance (2.2% to 13%; p = 0.009) and a reduction in the presence of ECAP responses (53.8% to 28%). The eSRT had a limited number of measurements, restricting the analysis.

**Conclusion:**

Electrode deactivation, when clinically indicated, did not compromise functional auditory performance, although it was accompanied by electrical and physiological changes. These findings reinforce the need for comprehensive monitoring and individualized management.

## INTRODUCTION

Cochlear implant (CI) electrode arrays, typically comprising 12 to 22 individual contacts, are designed to selectively stimulate different regions of the cochlea and the vestibulocochlear nerve. This design takes advantage of the tonotopic organization of the cochlea, assigning high-frequency information to basal electrodes and low-frequency information to apical electrodes, which is essential for speech sound perception^(1,2)^. Thus, activation of all fully inserted intracochlear electrodes aims to restore this critical tonotopic organization and promote effective stimulation of the auditory portion of the vestibulocochlear nerve^(3)^.

However, in clinical practice, electrical stimulation often excites broader regions than intended, resulting in overlapping neural excitation and increased electrode interaction. This phenomenon, known as channel interaction, has been associated with poorer speech perception outcomes in CI users^(4,5)^. The electrode–neuron interface and the fidelity with which natural hearing is simulated depend on several factors, including the precise position of the electrode within the cochlea, its distance from the modiolus, and the integrity of cochlear nerve fibers^(5,6)^. High levels of channel interaction may reduce spectral selectivity and limit the benefit provided by electrical stimulation^(4,7)^.

Programming strategies aimed at reducing channel interaction, including more focused stimulation and strategic selective deactivation of specific electrode channels, have shown potential to improve speech perception^(4,7)^. Imaging techniques, such as high-resolution computed tomography, allow visualization of the relationship between the electrodes and the cochlea and may guide individualized programming strategies^(6,7)^. However, these resources are not available in all centers and are not always sufficient to fully characterize the electrical behavior of an electrode over time or to assist in the diagnosis of electrode malfunction.

Situations in which the deactivation of one or more electrodes becomes necessary remain common in clinical practice. In many cases, deactivation is used to mitigate excessive channel interaction and increase stimulation independence by directing electrical energy toward contacts with a more favorable electrode–neuron interface^(6,8)^. In addition, deactivation may be indicated in cases of insertion trauma, extracochlear or nonfunctional electrodes, reduced dynamic range, discomfort at high stimulation levels, persistent patient complaints, and nonauditory sensations^(8)^. Studies suggest that strategic deactivation of contacts with suboptimal coding may improve sound-field audiometric thresholds and speech recognition performance, particularly under more complex listening conditions^(9-11)^.

The identification of electrodes with altered behavior can be performed both intraoperatively and during longitudinal follow-up. Impedance telemetry measures allow monitoring of abnormally high, low, or fluctuating patterns, which may indicate changes in the electrode–tissue interface^(12-14)^. Similarly, the absence of responses in electrically evoked compound action potentials (ECAPs) or electrically evoked stapedial reflex thresholds (eSRTs), when interpreted together with clinical findings suggestive of inadequate electrode function, may support the decision to deactivate a specific contact^(12,15)^. These objective measures are particularly relevant in patients who have difficulty reliably reporting their auditory perceptions.

In summary, optimization of speech perception in CI users depends on improving signal coding within the cochlea and minimizing electrode interaction. Suboptimal coding by specific contacts has been associated with poorer performance on speech recognition tests, and selective deactivation of these electrodes has been proposed as a strategy to refine the pattern of electrical stimulation^(9,10)^. Clinical and computational studies indicate that deactivation based on anatomical and functional criteria may preserve, or even improve, speech performance in adult CI users^(10,11)^.

Recent Brazilian studies, such as that by Danieli et al.^(11)^, have demonstrated the potential of image-based protocols to guide contact deactivation and improve speech performance, reinforcing the need for evidence integrating objective measures, imaging findings, and functional outcomes in real-world clinical settings.

Nevertheless, important gaps remain in the literature regarding the impact of electrode deactivation under routine clinical practice conditions, particularly in heterogeneous samples including different CI manufacturers, broad age ranges, and diverse audiological profiles. Questions also remain regarding when electrode deactivation is clinically necessary and its specific impact on users’ speech perception. Therefore, the present study aimed to determine whether electrode deactivation in cochlear implant users results in changes in speech recognition in quiet and in noise, as well as to identify whether changes occur in electrical and physiological measures.

## METHODS

This was a retrospective chart review conducted at the Cochlear Implant Section of a university rehabilitation center. The study was approved by the institutional Research Ethics Committee (CAAE: 63379022.8.0000.5441; approval number 5.788.962), with waiver of the requirement for informed consent. Cochlear implant (CI) users who fully met the established selection criteria were included. The inclusion criteria were: complete insertion of the electrode array confirmed by postoperative imaging; need for deactivation of at least one electrode during clinical follow-up; effective use of the device according to patient or family report; absence of sensory deprivation for a period equal to or greater than six months; and ability to perform open-set auditory recognition with the CI. Individuals with incomplete medical records, technical recording failures, insufficient experience with the implant, comorbidities that precluded the assessments, or inconsistencies between pre- and post-deactivation measures were excluded.

The procedures included analysis of device programming records using SoundWave 3.1® software (Advanced Bionics), Maestro 9.0® software (MED-EL), and Custom Sound EP 3.0® software (Cochlear). Information was collected regarding deactivated electrodes, intracochlear position, programming parameters, impedance values, electrically evoked compound action potential (ECAP) responses, and electrically evoked stapedial reflex threshold (eSRT) responses. The rationale for electrode deactivation followed the classification detailed in Appendix A^(12)^.

Audiological assessments were performed at two distinct time points: pre-deactivation, with all inserted electrodes active, and post-deactivation, after stabilization of the new map containing only the electrodes that remained active. The interval between assessments was not uniform among participants and varied widely according to each individual clinical schedule. Sound-field audiometry was performed in an acoustic booth using warble tones at 500, 1,000, 2,000, and 4,000 Hz, with the loudspeaker positioned 1 meter away at 0° azimuth. Sentence recognition testing was performed using recorded lists of Brazilian Portuguese sentences, presented in quiet and in competing “party noise” at 50 dB SPL (SNR +10 dB), and the final score was calculated according to the percentage of words correctly repeated.

Objective measures included: (1) impedance telemetry, with contacts classified as normal or altered, considering the presence of any altered electrode as a binary variable; (2) ECAP, with responses classified as present or absent, respecting the electrical compliance limits of each system; and (3) eSRT, with recording of the presence or absence of the stapedial reflex. In cases of absent neural responses due to compliance limits, pulse width was increased when technically feasible. The absence of responses in the pre-deactivation period precluded comparative analyses for eSRT.

The variables analyzed included age, sex, duration of auditory deprivation, duration of CI use, device manufacturer (Advanced Bionics®, Cochlear®, or MED-EL®), total number of deactivated electrodes, sound-field audiometric thresholds (dB HL), sentence recognition performance (% correct), impedance measures (normal/altered), ECAP responses (present/absent), eSRT responses (present/absent), and interval between assessments. Information on postoperative imaging examinations was also considered.

Subgroup comparisons, such as prelingual versus postlingual users, were not performed because of the small sample size in one of the groups and the risk of type II error. Statistical analyses were conducted considering the total sample, without stratification by linguistic profile, age group, or manufacturer, as the study was not designed for subgroup comparisons.

The significance level was set at 5% (p < 0.05). Considering the non-normal distribution of the data, the Wilcoxon signed-rank test for paired samples was used to compare pre- and post-deactivation performance in audiometric thresholds and speech tests. Spearman’s coefficient was applied to investigate the relationship between the number of deactivated electrodes, the interval between assessments, and auditory performance. The chi-square test was used to compare the proportions of contacts with altered impedance and the presence of ECAP responses. The results of the analyses are presented in Tables 1 to 5.

**Table 1:** Comparison of sound-field audiometric performance before and after electrode deactivation

	Before deactivation	After deactivation	P
500 Hz			
Median	30	27.5	0.540
Minimum	20	20	
Maximum	60	60	
1 kHz			
Median	30	27.5	0.034
Minimum	20	20	
Maximum	55	55	
2 kHz			
Median	30	25	0.241
Minimum	20	20	
Maximum	65	75	
4 kHz			
Median	30	30	0.094
Minimum	20	20	
Maximum	50	60	

**Table 5:** Comparison of objective measure results before and after electrode deactivation

Examination performed	Results	Pre	Post	p-value
Impedance telemetry	Any altered electrode	1 (2.2%)	6 (13.0%)	0.009^*^
	Normal Values	45 (97.8%)	40 (87.0%)	
eCAP	Responses present	7 (53.8%)	7 (28.0%)	0.156
	Responses absent	1 (7.7%)	9 (36.0%)	
	Any altered electrode	5 (38.5%)	9 (36.0%)	
eSRT	Reflexes present	7 (100%)	5 (62.5%)	N/A
	Reflexes absent	0 (100%)	3 (37.5%)	

* Statistically significant values according to the χ^2^ test

Caption: N/A indicates that hypothesis testing was not performed because there were zero observations in the pre-eSRT period

## RESULTS

### Sample characteristics

Among the 1,740 cochlear implant (CI) users followed at the service, 46 met the inclusion criteria for this study. The mean age was 24.7 years, ranging from 10 to 65 years, with a predominance of individuals with prelingual hearing loss (76.1%). The sample consisted of 20 female participants (43.5%) and 26 male participants (56.5%). Participants with prelingual hearing loss had a mean auditory deprivation duration of 6.71 ± 6.13 years, whereas those with postlingual hearing loss had a mean duration of 36.2 ± 17.9 years. The mean duration of CI use was 8.07 ± 4.99 years among participants with prelingual hearing loss and 5.56 ± 4.99 years among those with postlingual hearing loss. Regarding CI manufacturers in the sample, 14 participants (30.4%) used Advanced Bionics® devices, 12 (26.1%) used Cochlear® devices, and 20 (43.5%) used MED-EL® devices.

### Deactivated electrodes

The mean number of deactivated electrodes per participant was one electrode. This value corresponded to 6.25% of the total array in Advanced Bionics® devices, 4.54% in Cochlear® devices, and 8.33% in MED-EL® devices. The maximum number of deactivated electrodes was four in Advanced Bionics® and Cochlear® devices, and three electrodes in MED-EL® devices.

### Reasons for deactivation

The documented reasons for electrode deactivation were: absence of neural responses on ECAP (22.8%), perceived benefit reported by the patient (21.1%), bothersome nonauditory symptoms (19.3%), impedance problems (12.3%), programming anomalies (8.8%), absence of stapedial reflex (7.0%), unspecified reasons (5.3%), and presence of an extracochlear electrode (3.5%).

### Audiometric performance

Comparison of pre- and post-deactivation sound-field audiometric thresholds (Table 1) showed no statistically significant differences at 500 Hz and 2.000 Hz (p > 0.05). Although statistically significant differences were observed at 1.000 Hz and 4.000 Hz among the 46 users evaluated, these changes were not associated with clinically meaningful effects on overall mean performance or speech perception between pre- and post-deactivation assessments. These findings suggest that deactivation of one or more electrodes did not compromise overall auditory performance in this sample.

### Sentence recognition performance

Sentence recognition scores in quiet and in noise (Table 2) showed no significant differences between pre- and post-deactivation assessments, either in quiet (p = 0.188) or in noise at SNR +10 dB (p = 0.604). Thus, functional performance in speech tasks remained stable after deactivation.

**Table 2:** Comparison of sentence recognition performance in quiet and in noise before and after electrode deactivation

	Before deactivation	After deactivation	p
Sentence recognition in quiet			
Median	0.695	0.71	0.532
Minimum	0	0	
Maximum	100	100	
Sentence recognition in noise			
Median	0.45	0.35	0.518
Minimum	0	0	
Maximum	76	44	

### Correlation with the number of deactivated electrodes

Analysis of the correlation between the total number of deactivated electrodes and post-deactivation auditory performance (Table 3) revealed weak but statistically significant negative correlations for thresholds at 1.000 Hz (r = –0.289; p = 0.050) and 4.000 Hz (r = –0.325; p = 0.028). No significant correlations were observed for the remaining frequencies or speech tests (p > 0.05).

**Table 3:** Correlation matrix between post-deactivation auditory performance and the number of deactivated electrodes

Measure	Statistic	Value
Post-field 1KHz	Spearman	-0.289
	p-value	0.050^*^
Post-field 2KHz	Spearman	-0.210
	p-value	0.161
Post-field 4KHz	Spearman	-0.325
	p-value	0.028*
Post-field 500 Hz	Spearman	-0.237
	p-value	0.114
Post-sentences in quiet	Spearman	-0.153
	p-value	0.310
Post-sentences in noise	Spearman	-0.154
	p-value	0.305

Total number of deactivated electrodes analyzed: 46 users; mean = 1 electrode per participant; maximum = 4

* Statistically significant values according to Spearman’s correlation test

### Correlation with the interval between assessments

The time interval between pre- and post-deactivation assessments showed no significant correlation with audiometric thresholds or speech test performance (Table 4), indicating that the elapsed time between assessments did not influence auditory outcomes.

**Table 4:** Correlation between sentence recognition performance in quiet and in noise, post-deactivation sound-field thresholds, and the time interval in months

Assessments	Statistic	Time (months)	Value
Post-field 1 kHz	Spearman coefficient	16 (0–131)	-0.088
	p-value		0.592
Post-field 2 kHz	Spearman coefficient	16 (0–131)	-0.097
	p-value		0.559
Post-field 4 kHz	Spearman coefficient	16 (0–131)	-0.229
	p-value		0.161
Post-field 500 Hz	Spearman coefficient	16 (0–131)	-0.024
	p-value		0.883
Post-sentences in quiet	Spearman coefficient	16 (0–131)	0.031
	p-value		0.852
Post-sentences in noise	Spearman coefficient	16 (0–131)	-0.067
	p-value		0.685

Time (months) is presented as median (minimum–maximum) of the interval between assessments in the sample

### Objective measures

Evaluation of objective measures (Table 5) demonstrated a significant increase in the proportion of electrodes with altered impedance after deactivation, from 2.2% to 13.0% (p = 0.009). A reduction in the presence of ECAP responses was also observed, from 53.8% to 28.0%, although this difference was not statistically significant (p = 0.156). Analysis of eSRT was limited by the absence of responses in the pre-deactivation period, which precluded formal comparisons.

## DISCUSSION

This study investigated the impact of electrode deactivation, motivated either by subjective complaints or by objective findings of inadequate electrode function, on the auditory performance of cochlear implant (CI) users with devices from different manufacturers. The results demonstrated that deactivation of one or a limited number of electrodes did not compromise performance in speech recognition tests in quiet or in noise, corroborating previous findings indicating that selective channel deactivation can be implemented without functional detriment in certain clinical scenarios^(10,11,16)^. This suggests that strategic deactivation may be implemented without impairing functional hearing.

The literature shows that the electrode–neuron interface and the degree of channel interaction play a crucial role in speech perception^(4,7)^. Studies have shown that electrical stimulation may excite broader regions than desired, resulting in neural overlap and reduced spectral selectivity^(4,5)^. Strategic deactivation of electrodes presenting suboptimal coding, altered impedance, or inconsistent physiological responses may improve stimulation independence and reduce channel interference, thereby favoring speech signal processing and potentially improving auditory performance with the device^(6,9,10)^.

In the present study, although no significant changes in functional performance were observed after deactivation, relevant changes were identified in objective measures. There was a significant increase in the proportion of electrodes with altered impedance and a reduction in the presence of ECAP responses after deactivation. Although these changes were not directly reflected in poorer speech recognition, they suggest that deactivation may be associated with physiological adaptations or changes in the electrode–tissue interface, as previously described in the literature^(13,14,17,18)^.

The observed reduction in ECAP responses may be related both to the electrical compliance limitations of implant systems after deactivation and to changes in the physiological environment surrounding the electrode. At higher impedance levels, eliciting responses within the current delivery limits of the device may become difficult, which does not necessarily indicate complete absence of neural activity^(14,17,18)^. This finding reinforces the importance of interpreting objective measures within the context of the clinical presentation and the patient’s functional behavior.

The results are also consistent with the study by Danieli et al.^(11)^, which demonstrated that image-guided deactivation may improve auditory performance in postlingual users, particularly when contacts positioned unfavorably are removed from the map^(11)^. Although the present study did not use imaging to guide deactivation, both studies converge in indicating that removal of inadequate electrodes does not necessarily compromise functional performance and may represent an effective optimization strategy in selected cases.

On the other hand, some authors have reported that reducing the number of electrodes may negatively affect performance, especially when several contacts are deactivated or when the evaluated population has specific characteristics, such as young children with prelingual hearing loss^(19,20)^. These discrepancies in the literature reinforce that the impact of deactivation depends on multiple factors, including the number and location of deactivated contacts, manufacturer, processing strategy, etiological condition, user age, and previous level of auditory development.

In this study, small negative correlations were identified at 1,000 Hz and 4,000 Hz between the number of deactivated electrodes and post-deactivation sound-field thresholds. Although these correlations were statistically significant, their coefficients were low, suggesting limited clinical effect. This trend may reflect the importance of basal cochlear regions for processing high-frequency components of speech or may indicate that deactivation of multiple channels along specific tonotopic trajectories warrants further investigation.

The absence of differences in speech recognition may also be related to the test conditions, particularly the use of a favorable signal-to-noise ratio (+10 dB), which may reduce sensitivity for detecting subtle changes in performance. Studies using more challenging SNRs could better elucidate the impact of deactivation in more complex everyday listening situations.

Finally, the findings reinforce the importance of individualized programming approaches. The decision to deactivate an electrode should integrate objective measures, such as impedance, ECAP, and eSRT, with subjective complaints, auditory and nonauditory symptoms, intracochlear positioning, and functional performance. Optimized programming depends on the synthesis of these factors rather than on the isolated analysis of a single measure.

### Study limitations

This study has limitations that should be considered when interpreting the results. The retrospective design and relatively small sample size, although common in cochlear implant research, restrict the generalizability of the findings. Medical record review also resulted in missing information in some cases, which affected the completeness of certain variables.

The heterogeneity of the sample, including different age groups, pre- and postlingual profiles, and devices from different manufacturers, may have contributed to the variability of the results and limited the identification of more specific effects of electrode deactivation. In addition, speech recognition assessments were performed only under a moderately favorable listening condition (SNR +10 dB), which may not fully reflect users’ performance in more challenging environments.

Another important limitation was the absence of subjective measures, such as quality-of-life or user-perception questionnaires, which could have complemented the objective and functional measures and provided a more comprehensive view of the clinical impact of deactivation. Finally, the study was conducted at a single institution, which restricts the diversity of the evaluated profiles and reinforces the need for external validation in larger, multicenter samples.

Although the sample included pre- and postlingual users, different age groups, and multiple CI manufacturers, the study design did not include comparative analyses among these subgroups. The relatively small sample size within each stratum and the risk of type I and type II errors in unplanned multiple analyses justified the decision to focus interpretation on the overall results. Future studies with larger samples, specifically designed to compare pre- and postlingual profiles, different age groups, and manufacturers, are needed to clarify the impact of deactivation in these subgroups.

## CONCLUSION

Selective electrode deactivation did not compromise speech recognition in quiet or in noise in this sample, although it was associated with changes in electrical and physiological measures. These findings suggest that deactivation may be considered a valid clinical strategy in specific cases, provided that it is accompanied by objective monitoring. Future prospective studies with larger samples, integration of imaging examinations, and more challenging listening conditions are needed to define more robust criteria for this indication, further clarify the impact of deactivation on signal coding, and guide personalized cochlear implant programming protocols.

## Appendix A Classification of Signs and Symptoms for Electrode Deactivation

1. Unspecified

● Cause not determined.

2. Reduced Auditory Perception

● No loudness growth.

● Limited electrical dynamic range.

● Nonmeasurable auditory sensation.

● Difficulty distinguishing pitch.

● Sound perceived as softer.

● Extracochlear electrode.

3. Bothersome Auditory Symptoms

● Metallic sound.

● Distortion.

● Excessive background noise.

● Tinnitus.

● Minimal or very weak sound.

● Shrill or high-pitched sound.

● Bothersome high frequencies.

● Static sound.

● Tonal change toward tinnitus.

● Whistling.

● Different or altered sound.

● Discomfort with sound.

● Intolerable, painful, or uncomfortable sensation.

● Improvement in sound quality, clarity, and comfort after deactivation.

4. Bothersome Nonauditory Symptoms

● Discomfort before reaching the comfortable stimulation level, C-level.

● Extreme discomfort.

● Facial stimulation or contraction.

● Pulsation without auditory sensation.

● Dizziness.

● Eye movement.

● Nausea.

● Neck pain.

● Nasal tickling sensation.

● General physical pain.

● Sensation of pressure.

● Tactile sensations, including pulsating or vibrating sensations.

● Tingling.

● Headache.

● Intolerance to stimulation.

● Auditory sensation associated with nonauditory physical symptoms.

5. Software-Directed Inactivation

● Auto-deactivation in hybrid systems.

● Default inactivation.

● Specific features of hybrid programming.

6. Programming Abnormalities

● Compliance limitations.

● Downward electrode peaks.

● Elevated C, M, or TC levels.

● Elevated IFT.

● Open circuit.

● Out-of-compliance T levels.

● Poor sound quality associated with short circuit.

● Reduced dynamic range.

● Sawtooth response pattern.

● Short circuit, isolated or multiple.

● Low tolerance to stimulation with low M levels.

● Compliance restrictions at stimulation levels.

● Excessive stimulation.

7. Impedance Problems

● Elevated impedance.

● Compliance compromised by impedance changes.
