# Supplementary material for: Electrode deactivation in cochlear implant users: impact on speech perception and objective measures
Source: Codas. 2026 Jul 27;38(5):e20250290. doi: 10.1590/2317-1782/e20250290en (PMC13440451; doi:10.1590/2317-1782/e20250290en)
Supplement: Translation

# Translation: Desativação de eletrodos em usuários de implante coclear: impacto na percepção da fala e em medidas objetivas

**DOI:** 10.1590/2317-1782/e20250290pt

**Authors:** Carlos Alberto Conceição Santana, Júlia Speranza Zabeu Fernandes, Luiz Fernando Manzoni Lourençone

**Affiliations:** 1 Hospital de Reabilitação de Anomalias Craniofaciais – HRAC, Universidade de São Paulo – USP - Bauru (SP), Brasil.; 2 Seção de Implante Coclear, Hospital de Reabilitação de Anomalias Craniofaciais – HRAC, Universidade de São Paulo – USP - Bauru (SP), Brasil.; 3 Departamento de Otorrinolaringologia, Faculdade de Medicina de Bauru, Universidade de São Paulo – USP - Bauru (SP), Brasil.

**Keywords:** Audiologia, Perda Auditiva, Implante Coclear, Percepção da Fala, Potenciais Evocados, Reabilitação Auditiva, Eletrodos, Impedância Elétrica

## Abstract

**Objetivo:**

Verificar se a desativação de eletrodos em usuários de implante coclear resulta em modificações no reconhecimento de fala em silêncio e no ruído, bem como identificar se ocorrem alterações nas medidas elétricas e fisiológicas.

**Método:**

Estudo retrospectivo de prontuários de 46 usuários de implante coclear, de três fabricantes, submetidos à desativação de pelo menos um eletrodo. Foram analisados dados pré e pós-desativação referentes aos registros de programação, audiometria em campo livre (500, 1.000, 2.000 e 4.000 Hz) e testes de reconhecimento de sentenças gravadas em silêncio a 60 dB e com ruído competitivo a +10 dB de relação sinal-ruído. As medidas objetivas incluíram telemetria de impedância, reflexo estapediano eletricamente eliciado (eSRT) e potenciais de ação compostos evocados eletricamente (ECAP). As análises estatísticas utilizaram os testes de Wilcoxon, correlação de Spearman e qui-quadrado, com nível de significância de 5%.

**Resultados:**

Foram observadas correlações estatisticamente significativas nos limiares em 1.000 e 4.000 Hz nos 46 usuários estudados, porém sem repercussão funcional nas médias gerais ou na percepção de fala entre as avaliações pré e pós-desativação. As medidas objetivas mostraram alterações significativas, incluindo aumento de eletrodos com impedância alterada (2,2% para 13%; p = 0,009) e redução na presença de respostas de ECAP (53,8% para 28%). O eSRT apresentou número limitado de medidas, restringindo a análise.

**Conclusão:**

A desativação de eletrodos, quando clinicamente indicada, não comprometeu o desempenho auditivo funcional, embora tenha sido acompanhada de alterações elétricas e fisiológicas. Esses achados reforçam a necessidade de monitoramento abrangente e manejo individualizado.

## INTRODUÇÃO

As matrizes de eletrodos do implante coclear (IC), tipicamente contendo de 12 a 22 contatos individuais, são projetadas para estimular seletivamente diferentes regiões da cóclea e nervo vestíbulo coclear. Esse design aproveita a organização tonotópica coclear, direcionando frequências agudas para os eletrodos basais e frequências graves para os eletrodos apicais, o que é fundamental para a percepção dos sons de fala^(1,2)^. Assim, a ativação de todos os eletrodos totalmente inseridos na cóclea visa restaurar essa tonotopia crucial e promover uma estimulação eficaz da porção auditiva do nervo vestíbulo-coclear^(3)^.

Entretanto, na prática clínica a estimulação elétrica frequentemente excita áreas mais amplas do que o desejado, resultando em sobreposição da excitação neural e maior interação entre eletrodos. Esse fenômeno de interação de canais está associado a piores desfechos em percepção de fala em usuários de IC^(4,5)^. A interface eletrodo-neurônio e a fidelidade da simulação da audição natural dependem de fatores como a posição precisa do eletrodo na cóclea, a distância em relação ao modíolo e a integridade das fibras do nervo coclear^(5,6)^. Níveis elevados de interação entre canais podem reduzir a seletividade espectral e limitar o benefício da estimulação elétrica^(4,7)^.

Estratégias de programação que visam reduzir a interação de canais, incluindo estimulação mais focalizada e desativação seletiva estratégica de canais de eletrodos específicos, têm demonstrado potencial para melhorar a percepção de fala^(4,7)^. Técnicas de imagem, como tomografia computadorizada de alta resolução, possibilitam visualizar a relação entre eletrodos e cóclea e orientar estratégias de programação individualizadas^(6,7)^; entretanto, esses recursos não estão disponíveis em todos os centros e nem sempre são suficientes para caracterizar completamente o comportamento elétrico de um eletrodo ao longo do tempo e auxiliar no diagnóstico de mau funcionamento do eletrodo.

Situações em que a desativação de um ou mais eletrodos se torna necessária ainda são comuns na prática clínica. Em muitos casos, a desativação é utilizada para mitigar a interação excessiva entre canais e aumentar a independência da estimulação, direcionando a energia elétrica para contatos com melhor interface eletrodo-neurônio^(6,8)^. Além disso, a desativação pode ser indicada diante de trauma de inserção, presença de eletrodos extra cocleares ou não funcionais, faixa dinâmica reduzida, desconforto em altos níveis de estimulação, queixas persistentes do paciente e sensações extra auditivas^(8)^. Estudos sugerem que a desativação estratégica de contatos com codificação subótima pode melhorar limiares tonais da audiometria em campo livre e desempenho em reconhecimento de fala, especialmente em condições mais complexas de escuta^(9-11)^.

A identificação de eletrodos com comportamento alterado pode ser realizada tanto no momento intraoperatório quanto ao longo do seguimento do usuário. Medidas de telemetria de impedância permitem monitorar padrões anormalmente altos, baixos ou flutuantes, que são indicativos de alterações na interface eletrodo-tecido^(12-14)^. Do mesmo modo, a ausência de respostas em potenciais de ação compostos evocados eletricamente (ECAP) ou em limiares de reflexo estapediano eliciado eletricamente (eSRT), quando interpretada em conjunto com informações clínicas que indicam funcionamento inadequado, pode apoiar a decisão de desativar um ponto de contato específico^(12,15)^. Essas medidas objetivas são especialmente relevantes em pacientes com dificuldade de relatar de forma confiável suas percepções auditivas.

Em resumo, a otimização da percepção de fala em usuários de IC depende da melhoria da codificação do sinal dentro da cóclea e da minimização da interação entre eletrodos. A codificação subótima de determinados contatos está associada a pior desempenho em testes de reconhecimento de fala, e a desativação seletiva desses eletrodos tem sido proposta como uma estratégia para refinar o padrão de estimulação elétrica^(9,10)^. Estudos clínicos e computacionais indicam que a desativação baseada em critérios anatômicos e funcionais pode preservar ou até melhorar o desempenho de fala em adultos usuários de IC^(10,11)^.

Estudos nacionais recentes, como o de Danieli et al.^(11)^, têm demonstrado o potencial de protocolos baseados em imagem para orientar a desativação de contatos e melhorar o desempenho de fala, reforçando a necessidade de evidências que integrem medidas objetivas, achados de imagem e resultados funcionais em contextos clínicos reais.

Entretanto, ainda há lacunas importantes na literatura quanto ao impacto da desativação de eletrodos em condições de prática clínica rotineira, especialmente em amostras heterogêneas, que incluem diferentes fabricantes de IC, ampla faixa etária e variados perfis audiológicos. Ainda há dúvidas quanto ao momento em que a desativação do eletrodo é necessária, assim como o impacto em especial para percepção de fala do usuário. Diante desse cenário, o presente estudo tem como objetivo verificar se a desativação de eletrodos em usuários de implante coclear resulta em modificações no reconhecimento de fala em silêncio e no ruído, bem como identificar se ocorrem alterações nas medidas elétricas e fisiológicas.

## MÉTODO

Trata-se de uma análise retrospectiva de prontuários realizada na Seção de Implante Coclear de um centro universitário de reabilitação. O estudo foi aprovado pelo Comitê de Ética em Pesquisa da instituição (CAAE: 63379022.8.0000.5441; parecer nº 5.788.962), com dispensa do Termo de Consentimento Livre e Esclarecido. Foram incluídos usuários de implante coclear (IC) que atenderam integralmente aos critérios de seleção estabelecidos. Os critérios de inclusão foram: inserção completa da matriz de eletrodos confirmada por exame de imagem pós-operatório; necessidade de desativação de pelo menos um eletrodo durante o acompanhamento clínico; uso efetivo do dispositivo conforme relato do paciente ou familiares; ausência de privação sensorial por período igual ou superior a seis meses; e capacidade de reconhecimento auditivo em contexto aberto com o IC. Foram excluídos indivíduos com dados de prontuário incompletos, falhas técnicas de registro, tempo insuficiente de experiência com o implante, comorbidades que inviabilizassem as avaliações ou inconsistências entre as medidas pré e pós-desativação.

Os procedimentos incluíram a análise dos registros de programação dos dispositivos utilizando os softwares SoundWave 3.1® (Advanced Bionics), Maestro 9.0® (MED-EL) e Custom Sound EP 3.0® (Cochlear). Foram coletadas informações referentes aos eletrodos desativados, posição intracoclear, parâmetros de programação, valores de impedância, respostas do Potencial de Ação Composto Evocado Eletricamente (ECAP) e do reflexo estapediano eletricamente eliciado (eSRT). A justificativa para a desativação seguiu a classificação conforme detalhado no Apêndice A^(12)^.

As avaliações audiológicas foram realizadas em dois momentos distintos: pré-desativação, com todos os eletrodos inseridos ativos, e pós-desativação, após estabilização do novo mapa contendo somente os eletrodos mantidos ativos. O intervalo entre as avaliações não foi uniforme entre os participantes, variando amplamente conforme o cronograma clínico individual. A audiometria em campo livre foi realizada em cabine acústica, utilizando tom modulado (warble tone) nas frequências de 500, 1.000, 2.000 e 4.000 Hz, com o alto-falante posicionado a 1 metro de distância, em 0° de azimute. O teste de reconhecimento de sentenças foi aplicado utilizando listas gravadas de sentenças em português brasileiro, apresentadas em condições de silêncio e de ruído competitivo tipo “festa” a 50 dB SPL (SNR +10 dB), e a pontuação final variou conforme o percentual de palavras repetidas corretamente.

As medidas objetivas incluíram: (1) telemetria de impedância, com classificação dos contatos como normais ou alterados, considerando-se a presença de qualquer eletrodo alterado como variável binária; (2) ECAP, com respostas classificadas como presentes ou ausentes, respeitando os limites de conformidade elétrica dos sistemas; e (3) eSRT, com registro da presença ou ausência do reflexo estapediano. Em casos de ausência de resposta neural por atingir limites de conformidade, tentou-se ampliar a largura de pulso quando tecnicamente viável. A ausência de respostas no período pré-desativação impossibilitou análises comparativas para o eSRT.

As variáveis analisadas incluíram idade (anos), sexo, tempo de privação auditiva, tempo de uso do IC (anos), fabricante do dispositivo (Advanced Bionics®, Cochlear® ou MED-EL®), número total de eletrodos desativados, limiares de audiometria em campo livre (dB HL), desempenho nos testes de reconhecimento de sentenças (% de acertos), medidas de impedância (normal/alterada), respostas ECAP (presente/ausente), respostas eSRT (presente/ausente) e intervalo entre avaliações (meses). Também foram consideradas as informações sobre exames de imagem pós-operatórios (realizado ou não realizado).

Comparações entre subgrupos (ex.: pré-lingual vs pós-lingual) não foram realizadas devido ao tamanho amostral reduzido em um dos grupos e ao risco de erro tipo II. As análises estatísticas foram conduzidas considerando a amostra total, sem estratificação por perfil linguístico, faixa etária ou fabricante, uma vez que o estudo não foi delineado para comparações entre subgrupos.

O nível de significância adotado foi de 5% (p < 0,05). Considerando a distribuição não normal dos dados, o teste de Wilcoxon para amostras pareadas foi utilizado para comparar o desempenho pré e pós-desativação nos limiares audiométricos e nos testes de fala. O coeficiente de Spearman foi aplicado para investigar a relação entre o número de eletrodos desativados, o intervalo entre avaliações e o desempenho auditivo. O teste do qui-quadrado foi empregado para comparar as proporções de contatos com impedância alterada e a presença de respostas ECAP. Os resultados das análises estão apresentados nas Tabelas 1 a 5.

**Tabela 1:** Comparação do desempenho em audiometria de campo livre pré e pós-desativação de eletrodos

	Antes da desativação	Após a desativação	P
500 Hz			
Mediana	30	27,5	0,540
Mínimo	20	20	
Máximo	60	60	
1 kHz			
Mediana	30	27,5	0,034
Mínimo	20	20	
Máximo	55	55	
2 kHz			
Mediana	30	25	0,241
Mínimo	20	20	
Máximo	65	75	
4 kHz			
Mediana	30	30	0,094
Mínimo	20	20	
Máximo	50	60	

## RESULTADOS

### Características da amostra

Entre os 1.740 usuários de implante coclear (IC) acompanhados no serviço, 46 atenderam aos critérios de inclusão desse estudo. A idade média foi de 24,7 anos (variando de 10 a 65 anos), com predominância de indivíduos com perda auditiva pré-lingual (76,1%). A amostra foi composta por 20 participantes do sexo feminino (43,5%) e 26 do masculino (56,5%). Os participantes com perda auditiva pré-lingual apresentaram em média 6,71 ± 6,13 anos de privação auditiva, enquanto os com perda auditiva pós-linguais apresentaram 36,2 ± 17,9 anos. O tempo médio de uso do IC foi de 8,07 ± 4,99 anos nos participantes com perda auditiva pré-linguais e 5,56 ± 4,99 anos nos pós-linguais. Quanto aos fabricantes de IC da amostra, 14 participantes (30,4%) utilizavam dispositivos Advanced Bionics®, 12 (26,1%) Cochlear® e 20 (43,5%) MED-EL®.

### Eletrodos desativados

O número médio de eletrodos desativados por participante foi de um eletrodo. Esse valor corresponde a 6,25% da matriz total nos dispositivos Advanced Bionics®, 4,54% nos Cochlear® e 8,33% nos MED-EL®. O número máximo de eletrodos desativados foi de quatro nos dispositivos Advanced Bionics® e Cochlear®, e de três eletrodos nos dispositivos da MED-EL®.

### Motivos para a desativação

As razões documentadas para a desativação dos eletrodos foram: ausência de respostas neurais no ECAP (22,8%), benefício percebido pelo paciente (21,1%), sintomas não auditivos incômodos (19,3%), problemas de impedância (12,3%), anomalias de programação (8,8%), ausência de reflexo estapediano (7,0%), razões não especificadas (5,3%) e presença de eletrodo extracoclear (3,5%).

### Desempenho audiométrico

A comparação dos limiares da audiometria em campo livre pré e pós-desativação (Tabela 1) não mostrou diferenças estatisticamente significativas nas frequências de 500 Hz e 2.000 Hz (p > 0,05), por outro lado foram observadas correlações estatisticamente significativas nos limiares em 1.000 e 4.000 Hz nos 46 usuários estudados, porém sem repercussão funcional nas médias gerais ou na percepção de fala entre as avaliações pré e pós-desativação. Esses achados indicam que a desativação de um ou mais eletrodos não comprometeu o desempenho auditivo global desta amostra.

### Desempenho no reconhecimento de sentenças

Os escores de reconhecimento de sentenças em silêncio e em ruído (Tabela 2) não apresentaram diferenças significativas entre as avaliações pré e pós-desativação, tanto em silêncio (p = 0,188) quanto no ruído com SNR +10 dB (p = 0,604). Dessa forma, o desempenho funcional em tarefas de fala permaneceu estável após a desativação.

**Tabela 2:** Comparação do desempenho no reconhecimento de sentenças no silêncio e no ruído antes e após a desativação dos eletrodos

	Antes da desativação	Após a desativação	p
Reconhecimento de sentenças no silêncio			
Mediana	0,695	0,71	0,532
Mínimo	0	0	
Máximo	100	100	
Reconhecimento de sentenças no ruído			
Mediana	0,45	0,35	0,518
Mínimo	0	0	
Máximo	76	44	

### Correlação com o número de eletrodos desativados

A análise da correlação entre o número total de eletrodos desativados e o desempenho auditivo pós-desativação (Tabela 3) revelou correlações negativas fracas, porém estatisticamente significativas, nos limiares de 1.000 Hz (r= –0,289; p = 0,050) e 4.000 Hz (r= –0,325; p = 0,028). Nas demais frequências e nos testes de fala, não foram observadas correlações significativas (p > 0,05).

**Tabela 3:** Matriz de correlação entre o desempenho auditivo pós-desativação e o número de eletrodos desativados

Medida	Estatística	Valor
Pós_Campo 1KHz	Spearman	-0,289
	p-valor	0,050^*^
Pós_Campo 2KHz	Spearman	-0,210
	p-valor	0,161
Pós_Campo 4KHz	Spearman	-0,325
	p-valor	0,028*
Pós_Campo 500 Hz	Spearman	-0,237
	p-valor	0,114
Pós_Sentenças no Silêncio	Spearman	-0,153
	p-valor	0,310
Pós_Sentenças no Ruído	Spearman	-0,154
	p-valor	0,305

Número total de eletrodos desativados analisados: 46 usuários (média = 1 eletrodo por participante; máximo = 4)

* Valores estatisticamente significativos de acordo com o teste de correlação de Spearman

### Correlação com o intervalo entre avaliações

O intervalo de tempo entre as avaliações pré e pós-desativação não apresentou correlação significativa com os limiares audiométricos ou com o desempenho nos testes de fala (Tabela 4), indicando que o tempo decorrido entre as avaliações não influenciou os resultados auditivos.

**Tabela 4:** Correlação entre o desempenho no reconhecimento de sentenças no silêncio e no ruído, limiares de campo livre pós-desativação com o intervalo de tempo em meses

Avaliações	Estatística	Tempo (meses)	Valor
Pós_Campo 1KHz	Coeficiente de Spearman	16 (0–131)	-0,088
	p-valor		0,592
Pós_Campo 2KHz	Coeficiente de Spearman	16 (0–131)	-0,097
	p-valor		0,559
Pós_Campo 4KHz	Coeficiente de Spearman	16 (0–131)	-0,229
	p-valor		0,161
Pós_Campo 500 Hz	Coeficiente de Spearman	16 (0–131)	-0,024
	p-valor		0,883
Pós_Sentenças no Silêncio	Coeficiente de Spearman	16 (0–131)	0,031
	p-valor		0,852
Pós_Sentenças no Ruído	Coeficiente de Spearman	16 (0–131)	-0,067
	p-valor		0,685

Tempo (meses) apresentado como mediana (mín–máx) do intervalo entre avaliações na amostra

### Medidas objetivas

A avaliação das medidas objetivas (Tabela 5) demonstrou um aumento significativo na proporção de eletrodos com impedância alterada após a desativação (2,2% para 13,0%; p = 0,009). Observou-se também uma redução na presença de respostas ECAP (53,8% para 28,0%), embora sem significância estatística (p = 0,156). A análise do eSRT foi prejudicada pela ausência de respostas no período pré-desativação, impossibilitando comparações formais.

**Tabela 5:** Comparação dos resultados das medidas objetivas pré e pós-desativação dos eletrodos

Exames realizados	Resultados	Pre	Pós	p-valor
Telemetria de Impedância	Qualquer eletrodo alterado	1 (2,2%)	6 (13,0%)	0,009^*^
	Valores normais	45 (97,8%)	40 (87,0%)	
eCAP	Respostas presentes	7 (53,8%)	7 (28,0%)	0,156
	Respostas ausentes	1 (7,7%)	9 (36,0%)	
	Qualquer eletrodo alterado	5 (38,5%)	9 (36,0%)	
eSRT	Reflexos presentes	7 (100%)	5 (62,5%)	N/A
	Reflexos ausentes	0 (100%)	3 (37,5%)	

* Representa valores estatisticamente significativos de acordo com o teste χ^2^

**Legenda:** N/A indica que o teste de hipótese não foi realizado porque havia zero observações no período pré-eSRT

## DISCUSSÃO

Este estudo investigou o impacto da desativação de eletrodos, motivada por queixas subjetivas ou por achados objetivos de funcionamento inadequado do eletrodo, no desempenho auditivo de usuários de implante coclear (IC) de diferentes fabricantes. Os resultados demonstraram que a desativação de um ou de um número limitado de eletrodos não comprometeu o desempenho nos testes de reconhecimento de fala em silêncio ou no ruído, corroborando achados prévios que indicam que a desativação seletiva de canais pode ser implementada sem prejuízo funcional em determinados cenários clínicos^(10,11,16)^. Isso sugere que a desativação estratégica pode ser implementada sem prejuízo para a audição funcional.

A literatura mostra que a interface eletrodo-neurônio e o grau de interação entre canais desempenham papel crucial na percepção de fala^(4,7)^. Estudos apontam que a estimulação elétrica pode excitar regiões mais amplas do que o desejado, resultando em sobreposição neural e redução da seletividade espectral^(4,5)^. A desativação estratégica de eletrodos que apresentam codificação subótima, impedância alterada ou respostas fisiológicas inconsistentes pode melhorar a independência da estimulação e reduzir a interferência entre canais, favorecendo o processamento do sinal de fala, potencialmente melhorando o desempenho auditivo do usuário com o dispositivo^(6,9,10)^.

No presente estudo, apesar de não terem sido observadas mudanças significativas no desempenho funcional após a desativação, foram identificadas alterações relevantes nas medidas objetivas. Houve aumento significativo na proporção de eletrodos com impedância alterada e redução na presença de respostas ECAP após a desativação. Essas mudanças, embora não tenham se refletido diretamente em piora do reconhecimento de fala, sugerem que a desativação pode estar associada a adaptações fisiológicas ou alterações na interface eletrodo-tecido, como já descrito na literatura^(13,14,17,18)^.

A redução nas respostas ECAP observada pode estar relacionada tanto às limitações de conformidade elétrica dos sistemas de implante após a desativação quanto a alterações no ambiente fisiológico do eletrodo. Em níveis mais altos de impedância, pode ocorrer dificuldade de elicitação de respostas dentro dos limites de fornecimento de corrente do dispositivo, o que não necessariamente reflete ausência completa de atividade neural^(14,17,18)^. Esse achado reforça a importância de interpretar as medidas objetivas no contexto do quadro clínico e do comportamento funcional do paciente.

Os resultados também estão alinhados ao estudo de Danieli et al.^(11)^, que demonstrou que a desativação orientada por imagem pode melhorar o desempenho auditivo em usuários pós-linguais, especialmente quando contatos posicionados de forma desfavorável são removidos do mapa^(11)^. Embora o presente estudo não tenha utilizado imagem para direcionar a desativação, ambos os trabalhos convergem ao indicar que a retirada de eletrodos inadequados não necessariamente compromete o desempenho funcional e pode até representar uma estratégia eficiente de otimização em casos selecionados.

Por outro lado, alguns autores descrevem que a redução do número de eletrodos pode afetar negativamente o desempenho, especialmente quando vários contatos são desativados ou quando a população avaliada apresenta características específicas, como crianças pequenas com perda auditiva pré-lingual^(19,20)^. Essas divergências na literatura reforçam que o impacto da desativação depende de múltiplos fatores: número e localização dos contatos desativados, fabricante, estratégia de processamento, condição etiológica, idade do usuário e nível de desenvolvimento auditivo prévio.

Neste estudo, pequenas correlações negativas foram identificadas nas frequências de 1.000 Hz e 4.000 Hz entre o número de eletrodos desativados e os limiares de campo livre pós-desativação. Embora essas correlações tenham sido estatisticamente significativas, seus coeficientes foram baixos, sugerindo efeito clínico limitado. Essa tendência pode refletir a importância das regiões cocleares basais para o processamento de componentes de alta frequência da fala, ou indicar que a desativação de múltiplos canais em trajetórias tonotópicas específicas merece maior investigação.

A ausência de diferenças no reconhecimento de fala também pode estar relacionada às condições de teste, especialmente ao uso de uma relação sinal-ruído favorável (+10 dB), que pode reduzir a sensibilidade para detectar pequenas alterações no desempenho. Estudos utilizando SNRs mais desafiadoras poderiam elucidar melhor o impacto da desativação em situações de escuta cotidianas mais complexas.

Por fim, os achados reforçam a importância de abordagens individualizadas de programação. A decisão de desativar um eletrodo deve integrar medidas objetivas (impedância, ECAP, eSRT), queixas subjetivas, sintomas auditivos e extra-auditivos, posicionamento intracoclear e desempenho funcional. A programação otimizada depende da síntese desses fatores, e não da análise isolada de uma única medida.

### Limitações do estudo

Este estudo apresenta limitações que devem ser consideradas na interpretação dos resultados. O desenho retrospectivo e o tamanho relativamente pequeno da amostra, embora comuns na pesquisa em implante coclear, restringem a generalização dos achados. A análise de prontuários também resultou em lacunas de informação em alguns casos, o que impactou a completude de determinadas variáveis.

A heterogeneidade da amostra, incluindo diferentes faixas etárias, perfis pré- e pós-linguais e dispositivos de distintos fabricantes, pode ter contribuído para a variabilidade dos resultados e limitado a identificação de efeitos mais específicos da desativação de eletrodos. Além disso, as avaliações de reconhecimento de fala foram realizadas apenas em uma condição de escuta moderadamente favorável (SNR +10 dB), o que pode não refletir plenamente o desempenho dos usuários em ambientes mais desafiadores.

Outra limitação importante foi a ausência de medidas subjetivas, como questionários de qualidade de vida ou percepção do usuário, que poderiam complementar as medidas objetivas e funcionais e fornecer uma visão mais abrangente do impacto clínico da desativação. Por fim, o estudo foi conduzido em uma única instituição, o que restringe a diversidade de perfis avaliados e reforça a necessidade de validação externa em amostras maiores e multicêntricas.

Além disso, embora a amostra incluísse usuários pré- e pós-linguais, diferentes faixas etárias e múltiplos fabricantes de IC, o delineamento deste estudo não contemplou análises comparativas entre esses subgrupos. O tamanho relativamente reduzido da amostra em cada estrato e o risco de erros do tipo I e II em análises múltiplas não planejadas justificaram a opção por focar a interpretação nos resultados globais. Estudos futuros, com amostras maiores e delineados especificamente para comparação entre perfis pré- e pós-linguais, diferentes faixas etárias e fabricantes, são necessários para esclarecer o impacto da desativação nesses subgrupos.

## CONCLUSÃO

A desativação seletiva de eletrodos não comprometeu o reconhecimento de fala em silêncio ou no ruído nesta amostra, embora tenha sido associada a alterações em medidas elétricas e fisiológicas. Esses achados sugerem que a desativação pode ser considerada uma estratégia clínica válida em casos específicos, desde que acompanhada por monitoramento objetivo. Estudos prospectivos futuros com amostras maiores, integração de exames de imagem e condições de escuta mais desafiadoras são necessários para definir critérios mais robustos para essa indicação, além de esclarecer melhor o impacto da desativação na codificação do sinal e para orientar protocolos personalizados de programação do implante coclear.

## Apêndice A Classificação de Sinais e Sintomas para Desativação de Eletrodos

1. Não Especificado

● Causa não determinada.

2. Percepção Auditiva Diminuída

● Sem crescimento de volume.

● Faixa dinâmica elétrica limitada.

● Sensação auditiva não mensurável.

● Dificuldade em distinguir altura (pitch).

● Som percebido como mais suave.

● Eletrodo extracoclear.

3. Sintomas Auditivos Incômodos

● Som metálico.

● Distorção.

● Ruído de fundo excessivo.

● Zumbido.

● Som mínimo ou muito fraco.

● Som estridente ou agudo.

● Frequências altas incômodas.

● Som estático.

● Mudança tonal para zumbido.

● Assobio.

● Som diferente ou alterado.

● Desconforto com o som.

● Sensação intolerável, dolorosa ou desconfortável.

● Melhora na qualidade sonora, clareza e conforto com a desativação.

4. Sintomas Não Auditivos Incômodos

● Desconforto antes do nível de estimulação confortável (nível C).

● Desconforto extremo.

● Estimulação ou contração facial.

● Pulsação sem sensação auditiva.

● Tontura.

● Movimento dos olhos.

● Náusea.

● Dor no pescoço.

● Cólica nasal (cócegas).

● Dor física geral.

● Sensação de pressão.

● Sensações táteis (inclusive pulsantes ou com vibração).

● Formigamento.

● Dor de cabeça.

● Intolerância à estimulação.

● Sensação auditiva associada a sintomas físicos não auditivos.

5. Inativação Direcionada por Software

● Auto-desativação em sistemas híbridos.

● Inativação por padrão.

● Características específicas de programação híbrida.

6. Anormalidades de Programação

● Limitações de conformidade.

● Picos de eletrodos para baixo.

● Níveis C, M ou TC elevados.

● IFT elevado.

● Circuito aberto.

● T’s fora de conformidade.

● Má qualidade sonora associada a curto-circuito.

● Faixa dinâmica reduzida.

● Padrão de resposta em dente de serra.

● Curto-circuito (isolado ou múltiplos).

● Baixa tolerância à estimulação com níveis M baixos.

● Restrições de conformidade nos níveis de estimulação.

● Estimulação excessiva.

7. Problemas de Impedância

● Impedância elevada.

● Conformidade comprometida por alterações de impedância.
